# Tea consumption and prostate cancer: an updated meta-analysis

**DOI:** 10.1186/1477-7819-12-38

**Published:** 2014-02-14

**Authors:** Yi-wei Lin, Zheng-hui Hu, Xiao Wang, Qi-qi Mao, Jie Qin, Xiang-yi Zheng, Li-ping Xie

**Affiliations:** 1 Department of Urology, The First Affiliated Hospital, School of Medicine, Zhejiang University, Qingchun Road 79, Hangzhou 310003, Zhejiang Province, China

**Keywords:** Prostate cancer, Tea, Meta-analysis

## Abstract

**Objectives:**

Tea is supposed to have chemopreventive effect against various cancers. However, the protective role of tea in prostate cancer is still controversial. The aim of this study is to elucidate the association between tea consumption and prostate cancer risk by meta-analysis.

**Methods:**

A total of 21 published articles were retrieved via both computerized searches and review of references. Estimates of OR/RR for highest *versus* non/lowest tea consumption levels were pooled on the basis of random effect model or fixed effect model as appropriate. Stratified analyses on tea type, population and study design were also conducted.

**Results:**

No statistical significance was detected between tea consumption and prostate cancer risk in meta-analysis of all included studies (odds ratio (OR) = 0.86, 95% CI (0.69-1.04)). Furthermore, stratified analyses on population (Asian, OR = 0.81, 95% CI (0.55-1.08); non-Asian, OR = 0.89, 95% CI (0.72-1.07)) and tea type (green tea, OR = 0.79, 95% CI (0.43-1.14); black tea, OR = 0.88, 95% CI (0.73-1.02)) also yielded non-significant association. Only the case–control study subgroup demonstrated a borderline protective effect for tea consumption against prostate cancer (OR = 0.77, 95% CI (0.55-0.98)).

**Conclusion:**

Our analyses did not support the conclusion that tea consumption could reduce prostate cancer risk. Further epidemiology studies are needed.

## Review

### Introduction

Prostate cancer remains one of the most common cancers afflicting men today. It is one of the most frequently diagnosed malignancies among men in the world [1]. Although the etiology of this malignancy remains unclear, both hereditary and environmental components are considered to contribute to prostate carcinogenesis, with age, race, and family history being the only well-established risk factors [2]. Recently, ecologic studies have offered substantial evidence that dietary factors may play a role in the etiology of prostate cancer [3]. As one of the most common beverages consumed worldwide, tea has gained increasing attention.

Tea is generally consumed in the form of black (fermented), green (unfermented) and oolong (partially fermented), all of which originate from the leaves of the plant *Camellia sinensis*[4]. Cellular study and animal xenograft research have showed that tea or the active ingredient in tea, polyphenols, could afford protection against a variety of cancer types [5]. Numerous epidemiological evidences support a protective effect of tea towards cancers [6-8]. However, the results based on epidemiological studies on the association of tea consumption with prostate cancer risk were inconsistent. A previous meta-analysis conducted by Zheng et al. [9] suggested that green tea but not black tea may have a protective effect on prostate cancer, especially in Asian populations. However, most recent epidemiological studies since the previous meta-analysis revealed inconclusive results [10,11]. Even more, a recent cohort study of Scottish [12] revealed that men with higher intake of tea are at greater risk of developing prostate cancer. Thus, we aimed to update and quantitatively re-assess the association between tea consumption and the risk of prostate cancer by summarizing the results of latest published studies. As such, we intended to provide the best available evidence as to whether tea consumption can reduce prostate cancer risk.

### Materials and methods

#### Literature search

We initially identified publications in the PubMed database up to July 2013 using keywords ‘tea’ and ‘prostate cancer’. Additional studies were identified from references cited in retrieved articles. Eligible studies should fulfill all the following inclusion criteria: (1) studies evaluating the tea consumption and prostate cancer risk; (2) the consumption of the natural green tea product, not of green tea extracts or supplements, were recorded; (3) the outcome of interest should be an incidence of prostate cancer; (4) odds ratio (OR) or relative risk (RR) estimates with corresponding 95% CIs (or sufficient data allowing us to compute them) were provided. In studies with overlapping patients or controls, the latest study with the largest sample size should be included.

#### Data extraction

Two investigators independently extracted the name of the first author, the year of publication, the population in which the study was conducted, study design, adjusted effects estimates, exposure assessment and adjusted covariates. Disagreement was resolved by either discussion or the third-party resolution. Considering that prostate cancer was a rare disease, the RR was assumed approximately the same as OR, and the OR was used as the study outcome. If a study provided separate adjusted ORs for different tea types or age stratifications, we treated them as independent studies.

#### Statistical analysis

The adjusted ORs/RRs and the corresponding 95% CI for highest *versus* non/lowest tea consumption levels were pooled, as only those with highest tea consumption level were considered as habitual consumer. Between-study heterogeneity across the eligible comparisons was measured using the chi-square-based Q test and *I*-square test [13]. Heterogeneity was considered significant if *P* <0.05. According to the significance of heterogeneity, the data from a single study were combined using either the fixed effect or random effect models [14,15]. In addition, subgroup in terms of study population (Asian *vs.* non-Asian), type of tea (black *vs.* green) and study design (cohort *vs.* case–control) were stratified. The potential publication bias was assessed graphically by funnel plot and estimated statistically by both Begg’s and Egger’s test [16]. *P* <0.05 was considered significant for publication bias. In the overall study, sensitivity analysis would be performed to evaluate the effect of a single study contributing to overall summary and the Duval and Tweedie non-parametric ‘trim and fill’ method would be used to account for publication bias if any publication bias appeared to exist [17]. All statistical analyses were performed with STATA (version 8.0, StataCorp, College Station, TX, USA). All *P* values were two-sided.

### Results

#### Eligible studies

A total of 21 published articles reporting tea consumption and risk of prostate cancer satisfied the inclusion criteria and were included in our meta-analysis [10-12,18-35]. Five articles providing separate adjusted ORs for different tea types were treated as independent studies (Table 1). The article by Slattery et al. [20] providing age stratification were regarded as two studies. The characteristics of the included studies are shown in Table 1. The publication dates in this study ranged between 1989 and 2013. Among them, nine studies were cohort studies, while the remainders were all case–control studies, including 10 hospital-based case–control studies and nine population-based case–control studies. Based on population feature, 16 studies were conducted in Asian, while 11 were the results from non-Asian. Tea type were specified in 21 studies, including 11 studies for black tea, nine studies for green tea and one study for oolong tea. For the unspecified tea type information, tea type was assumed according to local tea drinking preference, that is, black tea was predominantly consumed in English-speaking countries [12].

**Table 1:** Characteristics of studies on tea consumption and prostate cancer risk

**Author, year**	**Design**	**Population, region**	**Tea type**	**Lowest consumption level**	**Highest consumption level**	**OR/RR (95% CI) for the highest *vs. *** **lowest level**
Severson, 1989 [18]	Cohort	Japanese, USA	Green	None	Ever	1.47 (0.99-2.19)
	Cohort	Japanese, USA	Black	None	Ever	0.83 (0.61-1.13)
Vecchia, 1992 [19]	HCC	Italian, Italy	NS	None	> = 1 cup/day	0.9 (0.5-1.7)
Slattery, 1993 [20]	PCC	American, USA	NS	None	>5 cups/week	1.06 (0.72-1.57)
	PCC	American, USA	NS	None	>5 cups/week	0.90 (0.59-1.36)
Jain, 1998 [35]	PCC	Candian, Canada	NS	None	>500 g/day	0.70 (0.50-0.99)
Villenecuve, 1999 [21]	PCC	Canadian, Canada	NS	None	> = 4 cups/day	1.1 (0.8-1.5)
Ellison, 2000 [22]	Cohort	Canadian, Canada	Black	None	>750 ml/day	1.03 (0.58-1.82)
Sharpe, 2002 [33]	PCC	Canadian, Canada	Black	<54 drink-years	>107 drink-years	2.0 (1.3-3.0)
Sonoda, 2004 [34]	HCC	Japanese, Japan	Green	<=1 cup/day	> = 10 cups/day	0.67 (0.27-1.64)
	HCC	Japanese, Japan	Black	none	> = 1 cup/day	1.51 (0.89-2.56)
Allen, 2004 [23]	Cohort	Japanese, Japan	Green	<1 time/week	> = 5 times/week	1.29 (0.84-1.98)
	Cohort	Japanese, Japan	Black	<2 times/week	amost daily	0.86 (0.47-1.59)
Jian, 2004 [24]	HCC	Chinese, China	Green	None	> = 5 g/day	0.10 (0.04-0.23)
Kikuchi, 2006 [25]	Cohort	Japanese, Japan	Green	<1 cup/day	> = 5 cups/day	0.85 (0.5-1.43)
Kurahashi, 2008 [26]	Cohort	Japanese, Japan	Green	<1 cup/day	> = 5 cups/day	0.90 (0.66-1.23)
Wu, 2009 [27]	HCC	Chinese, China	Green	None	Ever	0.52 (0.28-0.96)
	HCC	Chinese, China	Black	None	Ever	0.55 (0.23-1.31)
	HCC	Chinese, China	Oolong	None	Ever	0.73 (0.39-1.37)
Tyagi, 2010 [32]	PCC	Indian, India	NS	None	Ever	0.45 (0.21-0.97)
Ganesh, 2011 [31]	HCC	Indian, India	NS	None	Ever	0.7 (0.1-3.4)
Stefani, 2011 [30]	HCC	Uruguayan, Uruguay	Black	None	> = 7 cups/week	0.43 (0.22-0.81)
Berroukche, 2012 [10]	HCC	Algerian, Algeria	Green	<=1 cups/day	> cups/day	0.6 (0.3-1.1)
Montague, 2012 [11]	PCC	Chinese, Singapore	Green	None	> = 2 cups/day	0.95 (0.62-1.45)
	PCC	Chinese, Singapore	Black	None	> = 2 cups/day	1.17 (0.67-2.07)
Shafique, 2012 [12]	Cohort	Scottish, Scotland	Black	0-3 cups/day	> = 7 cups/day	1.50 (1.06-2.12)
Geybels, 2013 [29]	Cohort	Dutch, Netherland	Black	<=1 cup/day	> = 5 cups/day	0.97 (0.8-1.17)
Geybels, 2013 [28]	PCC	American, USA	Black	<=1 cups/week	> = 2 cups/day	0.63 (0.45-0.9)

For stratified analysis, the tea type in these studies with unspecified information will be assumed as black tea according to tea drinking preference in these regions.

HCC: hospital-based case-conrol study; NS: not specified; PCC: population-based case–control study.

#### Risk assessment

**Figure 1:**
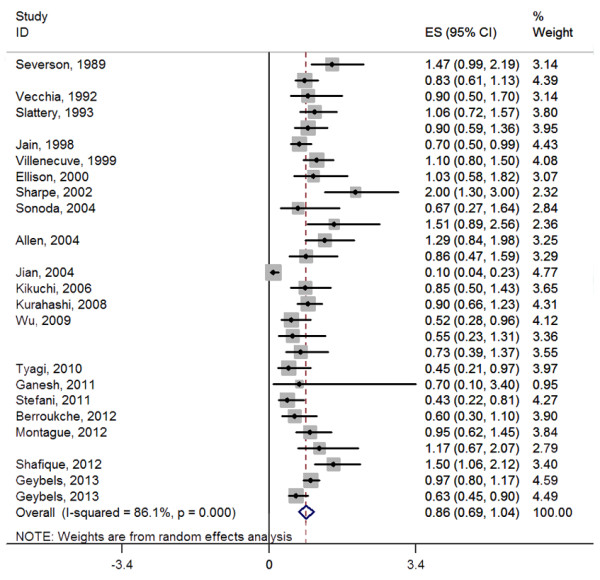
A forest plot showing risk estimates from 27 studies estimating the association between tea consumption and risk for prostate cancer.

**Figure 2:**
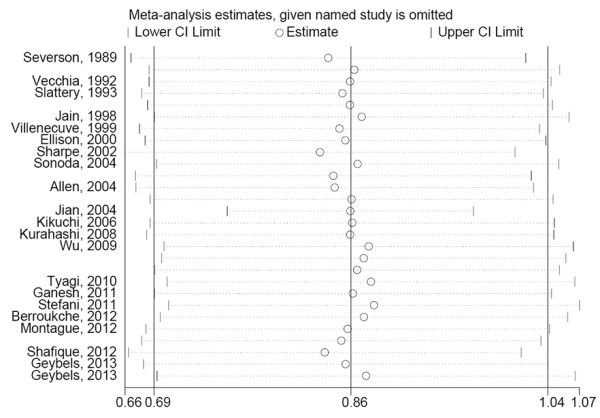
Sensitivity analysis demonstrates the influence of a single study to overall estimate.

The overall result, presented in Figure 1, showed no statistically significant association between tea consumption and prostate cancer (OR = 0.86, 95% CI (0.69-1.04)). Simultaneously, sensitivity analysis was performed to evaluate the effect of a single study on the overall estimate by sequentially excluding each study. We found that most studies could possibly not influence the overall risk estimate except the study by Jian et al. [24], which can offset the effect size to a border-line significance (OR = 0.86, 95% CI (0.75-0.98)) after being omitted (Figure 2).

**Figure 3:**
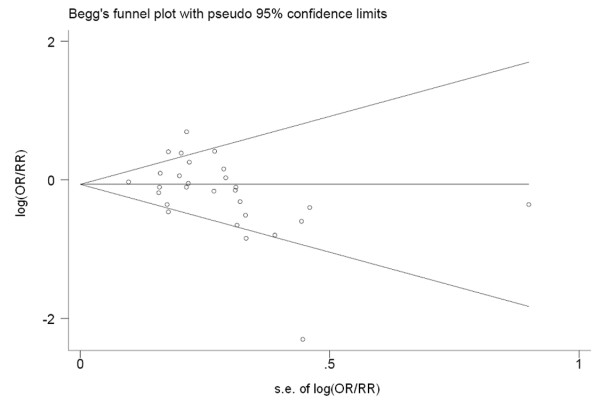
Begg’s funnel plot for publication bias of all studies on the association between tea consumption and prostate cancer risk.

For publication bias, visual exploration of the Begg’s funnel plot revealed an asymmetry shape (Figure 3). Further statistical test with Begg’s and Egger’s method indicated the suspicion of publication bias (*P*_Begg’s_ = 0.020, *P*_Egger’s_ = 0.066). But adjustment with ‘trim and fill’ method only yielded subtle variation (OR = 0.88, 95% CI (0.75-1.02)), which indicated that the conclusion from the overall summary was still reliable even though potential publication bias existed.

#### Stratified analyses

In the subgroup analyses (Table 2), the studies were stratified by population, tea type and study design. Considering characteristic disease trend [36] and tea drinking pattern [37], the population was stratified as Asian and non-Asian. In the population-stratified analysis, tea consumption did not reduce prostate cancer risk in both Asian and non-Asian (OR = 0.81 and 0.89, 95% CI (0.55-1.08) and (0.72-1.07), respectively). When stratified by tea type, neither the studies of green tea nor of black tea showed any significant protective effect of tea consumption in the prostate cancer risk (OR = 0.79 and 0.88, 95% CI (0.43-1.14) and (0.73-1.02), respectively). Since green tea was much more commonly drunk in the Asian population, we performed stratified analysis in depth. We found green tea consumption in the Asian population did not reduce prostate cancer risk (OR = 0.82, 95% CI (0.42-1.21)), as well as black tea in the non-Asian population (OR = 0.92, 95% CI (0.73-1.11)). When stratified by difference of study design, the summary of 18 case–control studies interestingly indicated a marginal protective effect of tea consumption against prostate cancer (OR = 0.77, 95% CI (0.55-0.98)), while the cohort studies did not show significant risk reduction (OR = 0.98, 95% CI (0.86-1.09)). Most stratified analyses did not present publication bias, except the Asian subgroup (*P*_Begg’s_ = 0.027) and green tea subgroup (*P*_Begg’s_ = 0.048, *P*_Egger’s_ = 0.041).

**Table 2:** Stratified analysis of tea consumption and prostate cancer risk

	**Studies (n)**	**OR (95% CI)**	**Test for heterogeneity**		**Test for publish bias**	
			***P***_**heterogeneity**_	***I***^***2***^**(%)**	***P***_**egger’s**_	***P***_**begg’s**_
All studies	28	0.86 (0.69-1.04)	<0.001	86.1	0.066	0.020
Region						
Asian	16	0.81 (0.55-1.08)	<0.001	86.3	0.071	0.027
Non-Asian	12	0.89 (0.72-1.07)	0.001	65.7	0.578	0.407
Tea type						
Green tea	9	0.79 (0.43-1.14)	<0.001	90.0	0.041	0.048
Black tea	18	0.88 (0.73-1.02)	0.002	56.6	0.499	0.272
Green tea in Asian	8	0.82 (0.42-1.21)	<0.001	91.0	0.068	0.063
Black tea in non-Asian	11	0.92 (0.73-1.11)	0.001	67.4	0.784	0.696
Study design						
Cohort studies	9	0.98 (0.86-1.09)	0.303	15.7	0.630	0.602
Case–control studies	19	**0.77 (0.55-0.98)**	<0.001	84.7	0.071	0.050

Significant results in bold.

### Discussion

Numerous studies have indicated daily tea consumption could be a non-invasive, economical and valuable means to prevent cancer. However, our current meta-analysis found that there was no significant association between tea consumption and reduced prostate cancer risk. Compared with the previous meta-analysis, our study has appended eight additional articles published after 2010 that were not included in the previous meta-analysis, which would strengthen the current conclusion.

Additionally, stratified analysis was performed according to population, tea type and study design in the studies to further verify our conclusion. Consistent with the overall summary, the most stratified analysis showed that there was no association between tea consumption and prostate cancer, except the summary of case–control studies which demonstrated a borderline inverse association between tea intake and the risk of prostate cancer. Also, it should be noted that both green tea and black tea consumption did not reduce prostate cancer risk, even though green tea was generally considered having a stronger anti-cancer effect.

The non-significant findings regarding the effects of tea consumption on prostate cancer in our meta-analysis contradicted the results of previous laboratory studies. Molecular study in cell-culture system and animal xenograft model research showed that both green tea polyphenol and black tea extracts [38-40] could afford anticancer potential towards prostate cancer. The compounds from tea could modulate carcinogenic signal pathway and induce apoptosis in prostate cancer cell. However, our meta-analysis failed to unveil the chemopreventive role of tea towards prostate cancer in epidemiological aspect. The contradiction was likely to be due to the lower quantities of human tea consumption compared to the doses used in experimental studies, while most cellular or animal xenograft studies used tea extract, such as catechins and flavonols, which had higher bioavailability. Also, the blood-prostate barrier could probably impair the penetration of active compounds from tea [41]. Therefore, the anti-cancer effect of tea would be weakened compared with cancer arising from the digestive tract. Besides, confounding effects of socioeconomic, dietary pattern and lifestyle factors probably masked the protective effect of tea consumption [42].

As is often the case with meta-analysis, several potential limitations in our study should also be taken into account. First, we did not attempt to include unpublished observations and studies with insufficient information to estimate an OR/RR, which probably would bias our results. Second, most included studies did not provided detail information on tea consumption duration or drinking pattern, for example, number of new batches brewed each day, quantity of tea leaves used per batch [24]. These parameters can influence the association between tea consumption and prostate cancer risk profoundly. Consequently, we did not have sufficient data to evaluate the risk of prostate cancer associated with these other dimensions of tea consumption. Third, even though we have used the maximal adjusted estimates, the adjusted criteria for OR/RR varied between studies. These probably would also bias our results.

## Conclusion

In summary, our current meta-analysis suggests that available evidences from this epidemiological study are insufficient to conclude that tea consumption could reduce the risk of prostate cancer. Further epidemiological studies are warranted to examine the relationships of tea and coffee with prostate cancer risk.

## Abbreviations

CI: Confidence interval; OR: Odds ratio; RR: Relative risk.

## Competing interests

The authors declare that they have no competing interests.

## Authors’ contributions

YL and ZH performed statistical analysis and wrote the manuscript; XW and QM performed literature search and stratified the data; JQ and XZ provided meaningful discussion key points; LX revised and edited the manuscript. All authors read and approved the final manuscript.
